# Prenatal maternal selenium plasma concentration and motor development in early infancy

**DOI:** 10.1017/S0007114526106485

**Published:** 2026-06-14

**Authors:** Suman Ranjitkar, Mari Hysing, Ram K. Chandyo, Tor A. Strand, Maria Averina, Manjeswori Ulak, Kjersti S. Bakken, Sandra Huber, Ingrid Kvestad

**Affiliations:** 1 Department of Psychosocial Science, Faculty of Psychology, University of Bergen, Bergen, Norway; 2 Department of Pediatrics, Child Health Research Project, Kathmandu, Nepal; 3 Community Medicine Department, Kathmandu Medical College, Kathmandu, Nepal; 4 Department of Research, Innlandet Hospital Trust, Lillehammer, Norway; 5 Center for International Health, University of Bergen, Bergen, Norway; 6 Department of Clinical Medicine, The Arctic University of Norway, Tromsø, Norway; 7 Department of Laboratory Medicine, University Hospital of North Norway, Tromsø, Norway; 8 Women’s Clinic, Innlandet Hospital Trust, Lillehammer, Norway

**Keywords:** Maternal selenium, Motor performance, Test of Infant Motor Performance, Infant, Nepal

## Abstract

Se is an important micronutrient that plays a key role in brain development. Only a few studies have explored the associations between prenatal maternal Se concentration and motor development in early infancy. We have previously described that 36 % of pregnant Nepalese women had Se concentration below the cut-off of 71·1 µg/l in early pregnancy. In the current cohort study, we aimed to describe the association between maternal plasma Se concentration and infant motor development measured at 8–12 weeks of age. From a cohort of 800 Nepalese mother–infant pairs, we included 711 dyads with available data on maternal Se concentration and motor development scores. Maternal Se concentration was measured in plasma samples collected within 15 weeks of gestation using inductively coupled plasma MS. Motor development was measured by the Test of Infant Motor Performance (TIMP). We examined the association between Se concentration and the TIMP scores in regression models adjusted for age of the mother and socioeconomic status. There was no association between maternal Se concentration and the TIMP scores (coefficient for the total TIMP score: −0·035 (95 % CI: −0·105, 0·036). In conclusion, even though a considerable proportion of the women had Se concentration below the cut-off of 71·1 µg/l, there was no association between maternal Se concentration and early motor development in their infants. Our findings do not support Se supplementation during pregnancy to enhance early infant motor development. However, Se may still be essential for other aspects of maternal and infant health.

## Graphical abstract

**Figure:**
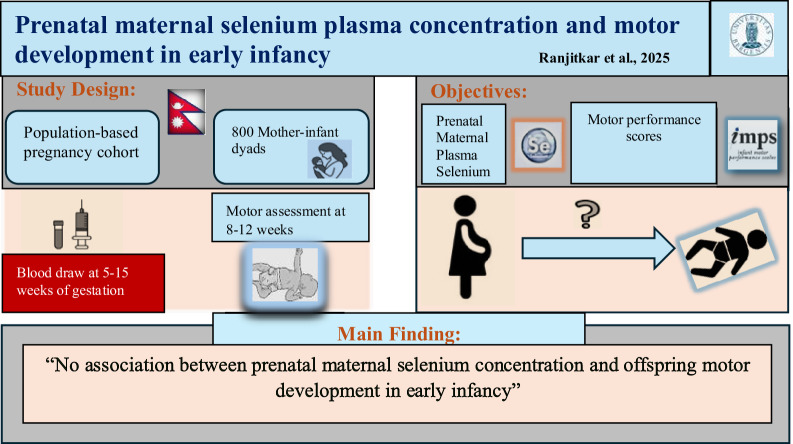

Prenatal brain development is primarily guided by genetic mechanisms influenced by the mother’s biochemical environment, such as nutritional status and exposure to toxins during pregnancy^(1)^. Though this dynamic process continues throughout a person’s life, the fetal brain’s heightened responsiveness to new experiences makes the prenatal period a time of great opportunity, but also vulnerability^(2)^.

Early motor development may be a sensitive outcome for prenatal exposures and also a predictor of later cognitive functioning in children^(3,4)^. The Test of Infant Motor Performance (TIMP) is an assessment tool of early motor development that may be performed to identify motor delays in infants^(5–7)^. In an overall evaluation of early motor assessment tools, the measurement qualities of the TIMP have been considered high, particularly in the youngest infants, with excellent predictive ability for future motor development^(8)^. We have previously found the tool to be reliable and feasible in a Nepalese context^(9)^.

Se is an important micronutrient for brain development and neurological function^(10,11)^. The demand for Se is increased in pregnant women, leading to a greater risk of Se deficiency^(12)^. The transport of Se within the body is retained by Selenoprotein-P in the blood, which plays a dynamic role in the brain, specifically in the cerebral and cerebellar cortices^(10,13)^. Both are closely related to overall motor functions as the length of the cerebellum of infants has shown associations with maternal Se concentration^(14)^, and the cerebellar cortex is mainly responsible for overall motor development^(15)^. Vitamin B_12_ may be relevant in the context of Se, as deficiency can disrupt one-carbon metabolism, and potential interactions between Se and vitamin B_12_ in pathways related to oxidative stress and neurodevelopment have been suggested, although evidence remains limited^(16–19)^.

Previous studies have shown associations between maternal prenatal Se concentration and child neurodevelopment across a wide range of domains, including motor development^(14,20–23)^. Studies have demonstrated positive associations between maternal Se concentrations and motor development in offspring during infancy and early childhood^(22–25)^. Contrasting with these studies, a study from Croatia showed no associations between prenatal maternal Se concentration and motor development using the Bayley Scales of Infant and Toddler Development, third edition^(14)^. To date, most studies examining these associations have focused on infants older than 6 months of age^(14,21–24)^. One exception is a study from China showing an inverted U-shaped relationship between umbilical cord blood Se concentration and neonatal neurobehavioral development assessed when the infants were 3 d old by the Neonatal Behavioural Neurological Assessment^(26)^. In this study, there was a negative association with higher Se concentration.

In a large mother–child cohort from Nepal, we found 36 % of the pregnant women were Se-deficient, but there was no association between Se concentration and neurodevelopment including motor development measured when the children were 6–24 months by the Bayley Scales of Infant and Toddler Development-third edition^(27)^. These pregnant women were originally included in a randomised controlled trial of the effect of vitamin B_12_ on neurodevelopment, where we detected no effect of B_12_ supplementation during pregnancy on the neurodevelopment at 6 and 12 months. There was, however, a negative effect of the intervention on early motor development as measured by TIMP when the children were 8–12 weeks^(28)^. These findings are interesting in the context of the association between maternal Se concentration and neurodevelopment, since there are previous studies that have described interaction effects of vitamin B_12_ in the association between maternal Se and neurodevelopment^(29)^. Sex has also been shown to have interaction effects in the association between maternal Se concentrations and motor development^(21)^. In a study from Bangladesh, higher Se concentrations were associated with improved psychomotor development in girls, but not in boys as measured by the Bayley Scales of Infant and Toddler Development-second edition when the children were 18 months old. Both maternal age and socioeconomic status may also potentially confound the association between maternal Se concentrations and infant neurodevelopment^(30–36)^.

The main objective of the present study is to describe the association between maternal Se concentration measured during early pregnancy and infant motor development measured by the TIMP at 8–12 weeks. In addition, we seek to assess the potential moderation by sex of the child and vitamin B_12_ supplementation.

## Method

### Study design and setting

The included mothers and infants were originally participants in a double-blinded randomised controlled trial entitled ‘Supplementation of vitamin B_12_ in pregnancy and postpartum on growth and neurodevelopment in early childhood: A Randomized, Placebo Controlled Trial’^(37)^. The study was conducted in Bhaktapur, located 15 km from Kathmandu, the capital city of Nepal. According to the census in 2021, Bhaktapur district has a population of 432 132 with an annual growth rate of 3·32 %^(38)^. People from this area are primarily engaged in agriculture, small-scale business, service or daily wage labour work.

Pregnant women aged 20–40 years, within 15 weeks of gestation and planning to stay in the study area for the next 2 years were enrolled. Gestation weeks were calculated based on the last menstruation period and re-confirmed by ultrasonography at enrolment. Pregnant women who were taking or planning to take multivitamins containing vitamin B_12_, with severe illnesses, high-risk pregnancies or a BMI outside the range of 18·5–30·0 kg/m^2^ were excluded from the study. The 800 pregnant women were randomly assigned to daily oral vitamin B_12_ (50 μg) or placebo supplementation from early pregnancy until 6 months postpartum. As per national guidelines, they were also supplemented daily with 400 µg of folic acid during the first trimester and from the second trimester to 45 d postpartum, with 500–1000 mg of calcium and 60 mg of iron.

**Figure 1.**
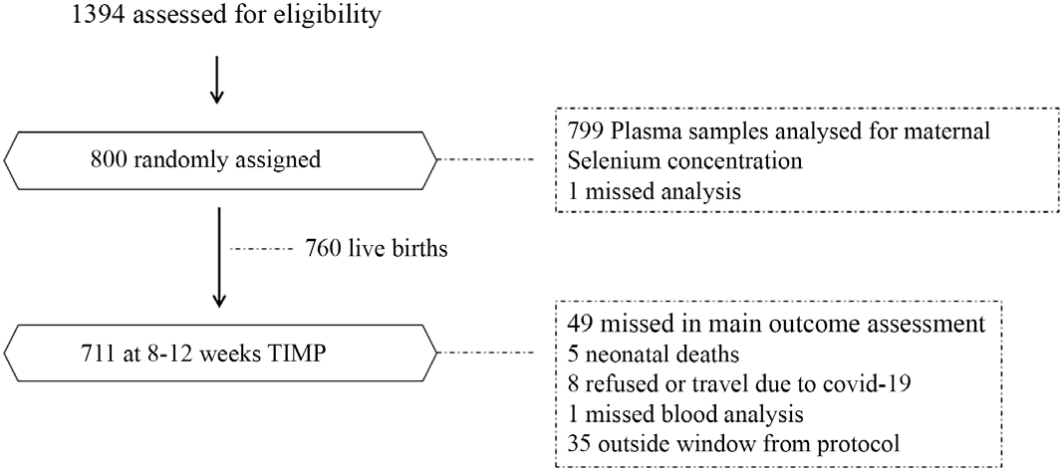
Study flow diagram for major activities from enrolment to 8–12 weeks in Nepalese mothers and children.

Pregnant women who met the eligibility criteria were enrolled from antenatal checkups from March 2017 to October 2020 and were followed every week until 6 months postpartum. When the infant was between 8–12 weeks of age, mother and child were requested to visit the study clinic for an assessment of motor performance. For the present study, we included mother–infant pairs for whom data were available on maternal Se concentration and TIMP scores within the specified assessment window period. Among 760 live births, five infants died, eight were not available due to refusal or travel during COVID-19 pandemic. A total of 740 infants were assessed using the TIMP, of which thirty-five were outside of the window period and were excluded, leaving 712 infants with valid TIMP assessments for the analysis. Among these, one participant lacked data on maternal Se concentration, resulting in a final sample size of 711. Details of the study flow are shown in Figure 1.

### Demographic and clinical information

At enrolment, demographic and clinical information were collected. Socioeconomic status was expressed using the Water and sanitation, Asset ownership, Maternal education, and household Income index, with scores ranging from 0–1^(39)^. The index was calculated based on baseline information provided by the participants on Water and sanitation, Asset ownership, Maternal education, and household Income^(39)^. Caste/ethnicity was recorded as Newar, Brahmin/Chhetri, Indigenous groups (Gurung/Rai/Magar, Tamang/Lama) and others (Chaudhari/Madhesi/Muslim, Dalits and others). Years of parental schooling were dichotomised as completed secondary school level (> 10 grade) and above or not. Parental occupation was dichotomised as work (engaged in agriculture, business, carpet factory, daily wage earning, government employment, services in the private sector and foreign employment) or no formal work. Maternal weight and height were measured at enrolment following the standard procedure with standard scales by trained field workers at a clinic using a portable electronic scale (Seca) and a stadiometer (Prestige, Hardik Medi Tech), respectively. BMI of mothers was calculated as body weight in kilograms and height in metre squared (kg/m^2^).

After delivery, information on gestational age and birth weight was obtained from hospital records. Those who were born < 37 weeks of the gestational period were defined as preterm, and birth weight < 2500 gm was considered low birth weight.

### Lab procedure and plasma selenium

EDTA blood samples (S-Monovette EDTA K3E, Sarstedt) were collected by trained laboratory technicians, centrifuged at 2000 G for 10 min and aliquoted into cryotubes for further storage at −70°C in the biobank. The plasma samples were shipped to Norway on dry ice for Se analysis.

Se was measured using inductively coupled plasma MS (ICP-MS, Nexion 300D, PerkinElmer) at the Environmental Pollutant Laboratory, Department of Laboratory Medicine, University Hospital of North Norway. 200 µl plasma samples were diluted by a liquid handler (1:20) with an alkaline reagent with helium as cell gas at 4·8 ml flow in kinetic energy discrimination mode. Rhodium (Inorganic Ventures) acted as an internal standard^(40)^. Matrix-matched calibration curves were prepared with clinical plasma samples (Recipe Chemicals and Instruments GmbH). All analyses were performed by qualified laboratory technicians according to the clinical accreditation standard NS-EN IS 15189:2012. The limit of detection for Se was 2·39 µg/l. Quality control samples from Recipe Chemicals, Germany, and Sero AS, Norway (ClinChek plasma and Seronorm serum, two levels each), were analysed together with each batch of 32 research samples and had coefficients of variation of 5 % and 6 %, respectively. All the quality controls were within the acceptance limits. The laboratory equipment was tested for background Se-contamination, and no significant contamination was found.

### Motor development

TIMP is a standardised test designed to assess the infant’s motor performance from 34 weeks postmenstrual age to 4–5 months^(5)^. TIMP is considered to be both a clinically and psychometrically sound tool for assessing early motor performance of infants^(41)^ and is widely used by physical and occupational therapists^(42)^. The test consists of forty-two items, of which thirteen are observed (dichotomised to zero-not observed and one-observed) and twenty-nine are elicited items (scores range from 0–6). The elicited items assess the infant activities such as head control, turning of the head or holding a stable posture, turning the head at the sound direction in prone position with neck and trunk extended, and crawling. The observed items assess spontaneous activities such as control of fingers and ankles, orientation of head in midline and development of ballistic, oscillating and fidgety movements.

Prior to the study, two assessors (a paediatrician and a psychologist) were trained in test procedures and administration. Standardisation procedures were conducted on twenty infants, during which both examiners scored the test simultaneously, yielding excellent interrater agreement (intraclass correlation coefficient > 0·94). Throughout the study, 5 % of the infants were randomly selected for double-scoring by the second assessor to ensure quality and consistency in test administration, yielding an intraclass correlation coefficient greater than 0·93^(9)^.

The assessments were performed in a room that was well-lit and free from distractions. We ensured that the infants were well-fed, healthy and not sleepy before starting the test. The test took approximately 20–50 min to administer. No adjustments for testing were considered for the current setting. Brief feedback on the infant’s performance was given if the infant was found delayed after assessment.

The total possible score for the TIMP is 142 (observed: thirteen and elicited: 129), with higher scores indicating better motor performance. The TIMP also offers age-specific norms based on a USA normative sample classifying the motor performance into average range (± 1 sd of the normative mean), low average (–0·5 sd to –1 sd of the normative mean), below average (−1 to −2 sd below the normative mean) and far below average range (< −2 sd below the normative mean)^(5)^.

### Statistical analysis

Statistical analyses were performed according to a predefined analysis plan registered at zenodo.org (https://zenodo.org/records/14523619). Demographic and clinical characteristics of the participant mothers are presented as means and sd and numbers (*n*) and percentages (%). We omitted any case that had missing data in any of the variables. With 290 participants in the Se-deficient group and 509 in the normal group, we had 80 % and 90 % power to detect standardised effect size differences of 0·21 and 0·24, respectively. These calculations were based on a two-sided *α* level of 0·05. Before the main analysis, we checked for curve linear associations between Se concentration and the TIMP scores through visual inspection (Stata command two-way fpfitci) and could not identify obvious curve linear relationships. The associations between the maternal Se concentration (continuous, dichotomised and tertiles) and the TIMP scores (total, observed and elicited) were estimated in multiple linear regression models adjusting for predefined potential confounders (i.e. maternal age and Water and sanitation, Asset ownership, Maternal education, and household Income index). Adjustment variables were set based on the literature that considered the variables as predictors both for maternal Se and motor development of infants^(30–36)^. We also examined the associations between the Se concentration and the total TIMP score dichotomised as scores below the average range (< –1 sd of the average norm scores) and within the average range (± 1 sd of the average norm scores) using logistic regression. We also ran all models, linear and logistic, with interaction terms between Se and child sex and Se and vitamin B_12_ treatment in separate models. Significance level was set at p-value <0·05. We used Stata version 18·5 for all analyses.

## Results

The average maternal age (sd) at enrolment was 27·6 (4·0) years. Of the 711 included women, around 68·2 % were working, and 79 % had completed secondary school or above. Altogether 66·1 % of the women were living in joint families, and 23·8 % were residing in rented houses and 26 % had kitchen and bed in the same room. Among the participants, 69·1 % had their own land, and 10·6 % received remittances from abroad (Table 1). Among the infants, 51·9 % were male, 9·3 % were born with low birth weight, and 8·6 % were born preterm (Table 2). The mean (sd) plasma maternal Se concentration was 74·8 µg/l (10·3 µg/l), and 35·7 % had Se deficiency using the cut-off of 71·1 µg/l. The mean (sd) TIMP total score when the infants were 8–12 weeks old was 77·1 (9·8), 317 of the infants (44·6 %) have TIMP scores below average.

**Table 1.** Demographic, socioeconomic and nutritional features of 711 pregnant mothers of Bhaktapur, Nepal Table 1 long description.

Demographic and socioeconomic features	*n* 711	
	*n*	%
Mother’s age		
Mean	27·6	
sd	4·0	
Education		
Mothers who completed secondary school or above	562	79·0 %
Fathers who completed secondary school or above	537	75·5 %
Occupation		
Mothers who work	485	68·2 %
Fathers who work	705	99·2 %
Caste/Ethnicity		
Newar	562	79 %
Brahmin /Chhetri	70	9·9 %
Indigenous group	61	8·6 %
Others	18	2·5 %
Socioeconomic status		
Monthly income^†^		
High (above 75k)	57	8·0 %
Medium (above 25k–75k)	377	53·0 %
Low (Up to 25k)	277	39·0 %
Family staying in joint family	470	66·1 %
Family residing in rented house	169	23·8 %
Kitchen and bedroom in same room	185	26 %
Family having own land	491	69·1 %
Receiving remittance from abroad	75	10·6 %
	Mean	sd
WAMI* (*n* 707)	0·66	0·13
Nutritional status of mother at enrolment		
Weight (kg)	55·1	7·5
Maternal BMI (kg/m^2^)	23·7	3·0
Micronutrient status of mother: Se and Hb concentrations		
Plasma Se concentration (µg/l) (*n* 711)		
Mean (sd)	74·8	10·3
	*n*	%
Low (<71·1 µg/l)	254	35·7 %
Normal (≥71·1 µg/l)	457	64·3 %
Median	74·3	
2·5th percentile	56·2	
5th percentile	58·7	
25th percentile	67·8	
75th percentile	80·7	
95th percentile	91·5	
97·5th percentile	97·5	
	Mean	sd
Lowest tertile (*n* 234)	63·9	4·7
Middle tertile (*n* 239)	74·3	2·2
Highest tertile (*n* 238)	86·1	6·9
Plasma Se concentration in infants with TIMP score below average (*n* 394)	74·9	10·5
Plasma Se concentration in infants with TIMP score within average (*n* 317)	74·8	10·1
Plasma Se concentration enrolled at gestational week up to 12 weeks (*n* 409)	75·9	10·3
Plasma Se concentration enrolled at gestational week after 12 weeks (*n* 302)	73·4	10·2
Hb concentration (g/dl) (*n* 710)	12·8	1·2

TIMP; Test of Infant Motor Performance.

All numbers are numbers (percentage) if not otherwise stated, ^†^Nepalese Rupees, *Water and sanitation, household assets, maternal education and household income, range 0–1.

**Table 2.** Characteristics and motor development of 711 infants in Bhaktapur, Nepal Table 2 long description.

Infant’s characteristics	*n*	%
Male	369	51·9 %
Low birth weight (< 2500 gm)	66	9·3 %
Pre-term birth (< 37 weeks of gestation)	61	8·6 %
	Mean	sd
TIMP total score	77·1	9·8
TIMP Observed score	8·1	1·3
TIMP Elicited score	69·0	9·5
	*n*	%
TIMP scores below average	317	44·6 %

TIMP, Test of Infant Motor Performance.

Among infants with TIMP scores below (*n* 394) and within (*n* 317) the average mean (sd), maternal Se concentration was 74·9 µg/l (10·5 µg/l) and 74·8 µg/l (10·1 µg/l), respectively. Maternal Se concentrations at gestational week up to 12 weeks and after were 75·9 µg/l (10·3 µg/l) and 73·4 µg/l (10·2 µg/l), respectively (Table 1).

The association between maternal plasma Se concentration and TIMP scores is present in Tables 3–5. There were no linear associations between maternal Se concentration and the TIMP total and subscale scores (*β* for the total TIMP score: −0·035 (95 % CI: adjusted −0·105, 0·036) (Table 3), and no association between maternal Se concentration and TIMP scores dichotomised as below and within average range (*OR* = 0·999 (95 % CI: adjusted −0·986, 1·014) (Table 5). When dichotomising the maternal Se concentration into deficient *v*. normal (Table 3) and categorising in tertiles (Table 4), there were no associations between Se concentration and the TIMP scores. Our analyses did not identify significant effect modifications by sex of the child and vitamin B_12_ supplementation across any of the models including analyses with TIMP scores as continuous and dichotomised and Se with continuous, dichotomised and categorised in tertiles (all *P*-values were above 0·05).

**Table 3.** Associations between maternal plasma selenium concentration and TIMP scores in 711 infants from Bhaktapur, Nepal Table 3 long description.

		Crude			Adjusted		
TIMP scores	Se exposure	Coeff.	95 % CI	*P*	Coeff.	95 % CI	*P*
TIMP total	Continuous	−0·035	−0·105, 0·035	0·33	−0·035	−0·105, 0·036	0·33
	Deficient*	Ref.			Ref.		
	Normal	−0·035	−1·544, 1·474	0·96	0·037	−1·479, 1·553	0·96
TIMP Observed	Continuous	−0·006	−0·015, 0·003	0·18	−0·006	−0·015, 0·003	0·19
	Deficient	Ref.			Ref.		
	Normal	0·095	−0·101, 0·292	0·34	0·103	−0·094, 0·300	0·31
TIMP Elicited	Continuous	−0·028	−0·096, 0·039	0·41	−0·029	−0·096, 0·039	0·41
	Deficient	Ref.			Ref.		
	Normal	−0·130	−1·586, 1·325	0·86	−0·066	−1·529, 1·398	0·93

TIMP, Test of Infant Motor Performance.

*Se concentration < 71·1 μg/l. Adjusted for maternal age and Water and sanitation, Asset ownership, Maternal education, and household Income. Analyses are based on linear regression models.

**Table 4.** Associations between maternal plasma selenium concentration (tertiles) and TIMP scores in 711 infants from Bhaktapur, Nepal Table 4 long description.

		Crude			Adjusted		
TIMP scores	Se concentration	Coeff.	95 % CI	*P*	Coeff.	95 % CI	*P*
TIMP total	Lowest tertile	−0·136	−1·910, 1·639	0·88	−0·039	−1·823, 1·745	0·97
	Middle tertile*	Ref.			Ref.		
	Highest tertile	−0·360	−2·127, 1·406	0·69	−0·326	−2·103, 1·452	0·72
TIMP Observed	Lowest tertile	0·074	−0·157, 0·305	0·53	0·085	−0·146, 0·317	0·47
	Middle tertile*	Ref			Ref.		
	Highest tertile	−0·067	−0·297, 0·163	0·57	−0·057	−0·288, 0·174	0·63
TIMP Elicited	Lowest tertile	−0·209	−1·922, 1·502	0·81	−0·124	−1·847, 1·598	0·89
	Middle tertile*	Ref.			Ref.		
	Highest tertile	−0·293	−1·998, 1·412	0·74	−0·269	−1·985, 1·447	0·76

TIMP, Test of Infant Motor Performance.

Adjusted for maternal age and Water and sanitation, Asset ownership, Maternal education, and household Income, *Reference group.

Analyses are based on linear regression models.

**Table 5.** Associations between maternal plasma selenium concentration and below average and average TIMP scores in 711 infants from Bhaktapur, Nepal Table 5 long description.

		Crude			Adjusted		
TIMP scores	Se concentration	OR	95 % CI	*P*	OR	95 % CI	*P*
Below average		0·999	0·985, 1·014	0·94	0·999	0·986, 1·014	0·99

TIMP, Test of Infant Motor Performance.

Adjusted for maternal age and Water and sanitation, Asset ownership, Maternal education, and household Income, Analyses are based on logistic regression models.

## Discussion

In this sample of Nepalese mother–infant dyads, a substantial proportion of the women were Se-deficient as measured within 15 weeks of gestation. The current study aimed to investigate the association between maternal Se concentration and motor performance measured in early infancy. Although Se plays a vital role in motor functions^(43,44)^ and there is a described maternal deficiency in the population, we could not find associations between maternal Se concentration and infants’ motor performance measured at 8–12 weeks of age in these Nepalese infants.

To our knowledge, this is one of very few studies to investigate the association between maternal Se concentration and infants’ motor performance. We have been able to identify one previous study from China that also measured early development^(26)^; however, findings from this study contrasted with our results showing an association between Se concentration and early development. The differences may be due to methodological issues. In the previous study, they used umbilical cord blood from neonates to measure Se concentration and motor development was measured by the Neonatal Behavioural Neurological Assessment scale. The Neonatal Behavioural Neurological Assessment is a broader scale not specifically designed to measure motor development^(45),^ and it was conducted when the children were only three days old^(26)^. Another key difference is the use of cord blood Se plasma concentration as a biomarker which might differ from Se concentration measured during the prenatal period. There are also other studies that showed significant positive associations between Se and motor development^(22–25)^. These assessed motor performance after 6 months of age; however, and in contrast to our study, most have measured the maternal Se concentrations in later pregnancy (i.e. during second and third trimester) or after delivery using umbilical cord blood^(22,23,26)^.

We could not find effect modifications of vitamin B_12_ in the association between maternal Se concentration and motor development of infants. Our findings also do not signify sex as a moderating factor in the association between maternal Se and motor development, that is discrepant from the findings from Bangladesh and China showing protective effects of Se in girls^(21,46)^. This could be because of relatively narrow safe range of Se concentrations required for optimum enzyme functioning among the participating mothers in our study^(47)^ showing neither very high nor very low maternal Se concentration, which may result in limited ability to detect sex-specific effect modifications in motor performance of the infants. Moreover, the null findings could be due to the lack of severely Se-compromised conditions in our sample, which might otherwise have led to neurodegeneration due to the competition between brain and testes for Se consumption in males^(48)^. Other reasons for the null findings could be attributed to the presence of multiple factors influencing neurodevelopment in high-risk groups, particularly in low-income and middle-income countries. These factors may overshadow or counteract the effects of Se, even though a significant proportion of individuals in the study was classified as Se-deficient based on the defined threshold (< 71·1 µg/l). Interaction between Se and other micronutrients has also been reported for various clinical outcomes. For example, higher folate status in combination with high Se has been associated with an increased risk of autism spectrum disorder^(29)^. In addition, interactions between Se and iron status have been suggested, whereby adequate Se intake may protect against reactive oxygen species in conditions of iron overload, such as secondary haemochromatosis^(49)^ and calcium has also shown interactions with Se in the improvement of skeletal muscle activity in mice^(50)^. In the current study, all mothers, regardless of study group, received folic acid, iron and calcium. We were, therefore, unable to examine the potential effect modification by these supplements. The questions on interactions between baseline maternal micronutrient biomarker concentration and maternal Se concentration in relation to neurodevelopmental outcomes should be raised in future studies.

About 45 % of the infants in the current study fall into the below average category of TIMP scores when using USA norms. This indicates that many of the children have a delay in motor development compared with the USA normative sample. According to our results, however, Se deficiency in pregnancy does not account for this developmental delay. In the current study population, there are many risk factors that may limit development in the children, such as low level of knowledge about child development among Nepalese mothers^(51)^, leading to a risk of limited exposure to learning materials and stimulation at home. Being born with low birth weight and length-for-age, preterm birth, anaemia and diet patterns are other prevalent risk factors that have been shown to be related to child development in this setting^(52,53)^. Another potential factor could be the short stature of mothers which have shown negative associations with cognitive and motor development in low-income and middle-income countries^(54)^. In other words, the null findings of our study could indicate that maternal Se concentration might not be a key factor of motor development in early infancy in the current population.

The strengths of the present work include a relatively large sample of up to 800 mother–infant dyads from a general Nepalese population and the high-quality measurement of motor development^(9)^. Laboratory procedures were carried out by trained and qualified technicians. Blood samples were stored and shipped from the study site in Bhaktapur to the laboratory in Norway in accordance with established biobanking standards. Moreover, we used Se concentration both as a continuous and categorical (deficient *v*. normal and in tertiles) variables in the regression models. Likewise, we assessed the associations using the motor scores both on a continuous scale and dichotomised according to USA norms. There are some limitations of this study. Plasma Se concentration might not be the ideal biomarker for Se status leading to random misclassification, and thus, both under- and overestimations of the association between maternal Se and motor performance could occur. Yet, we see that previous studies have found associations using the same biomarker^(22,24)^. The current cohort is from a healthy population participating in a clinical trial on the effect of vitamin B_12_ with specific exclusion criteria such as the requirement of adequate nutritional status. Generalisability of the results should therefore be cautiously handled.

### Conclusion

Despite Se deficiency being prevalent in the current study sample, we could not identify associations between maternal Se concentration and infant motor performance in a healthy cohort of Nepalese pregnant women. The findings could indicate that Se alone might not be a key factor of motor development in early infancy. Future research is suggested for an update of the nutrition recommendations for pregnant women and to better understand the specific contributing factor of early motor development.

## Table 1 Long description

The table presents demographic, socioeconomic, and nutritional features of 711 pregnant mothers in Bhaktapur, Nepal. It has 30 rows and 4 columns. The columns are labeled 'n 711', 'n', and '%'. The table is divided into several sections: Demographic and socioeconomic features, Nutritional status of mother at enrolment, Micronutrient status of mother, Plasma Se concentration, and Plasma Se concentration in infants. Each section contains specific data points. For example, under Demographic and socioeconomic features, the table lists the mean mother's age as 276 years with a standard deviation of 40 years. It also includes data on education, occupation, caste/ethnicity, socioeconomic status, and family living conditions. The Nutritional status section provides information on weight and maternal BMI. The Micronutrient status section includes plasma Se concentration levels. The Plasma Se concentration section categorizes Se concentration into low, normal, and highest tertile. The table provides detailed data on each of these categories, including mean, median, and various percentiles.

Navigate back to Table 1.

## Table 2 Long description

The table presents data on infant characteristics and TIMP scores. It has 8 rows and 3 columns. The columns are labeled Infant's characteristics, n, and %. Row 1: Male, 369, 51.9 percent. Row 2: Low birth weight (< 2500 grams), 66, 9.3 percent. Row 3: Pre-term birth (< 37 weeks of gestation), 61, 8.6 percent. Row 4: TIMP total score, Mean, 77.1. Row 5: TIMP Observed score, Mean, 8.1. Row 6: TIMP Elicited score, Mean, 69.0. Row 7: TIMP scores below average, 317, 44.6 percent.

Navigate back to Table 2.

## Table 3 Long description

A table comparing maternal plasma selenium concentration and TIMP scores in 711 infants from Bhaktapur, Nepal. The table has 10 rows and 10 columns. Column headers are TIMP scores, Se exposure, Crude Coeff., Crude 95 % CI, Crude P, Adjusted Coeff., Adjusted 95 % CI, Adjusted P. Row labels are TIMP total Continuous, TIMP total Deficient, TIMP total Normal, TIMP Observed Continuous, TIMP Observed Deficient, TIMP Observed Normal, TIMP Elicited Continuous, TIMP Elicited Deficient, TIMP Elicited Normal. Row 1: TIMP total, Continuous, -0.035, -0.105, 0.035, 0.33, -0.035, -0.105, 0.036, 0.33. Row 2: TIMP total, Deficient, Ref., Ref., Ref., Ref., Ref., Ref., Ref., Ref.. Row 3: TIMP total, Normal, -0.035, -1.544, 1.474, 0.96, 0.037, -1.479, 1.553, 0.96. Row 4: TIMP Observed, Continuous, -0.006, -0.015, 0.003, 0.18, -0.006, -0.015, 0.003, 0.19. Row 5: TIMP Observed, Deficient, Ref., Ref., Ref., Ref., Ref., Ref., Ref., Ref.. Row 6: TIMP Observed, Normal, 0.095, -0.101, 0.292, 0.34, 0.103, -0.094, 0.300, 0.31. Row 7: TIMP Elicited, Continuous, -0.028, -0.096, 0.039, 0.41, -0.029, -0.096, 0.039, 0.41. Row 8: TIMP Elicited, Deficient, Ref., Ref., Ref., Ref., Ref., Ref., Ref., Ref.. Row 9: TIMP Elicited, Normal, -0.130, -1.586, 1.325, 0.86, -0.066, -1.529, 1.398, 0.93.

Navigate back to Table 3.

## Table 4 Long description

A table comparing the association between maternal plasma selenium concentration and TIMP scores in 711 infants from Bhaktapur, Nepal. The table has 10 rows and 9 columns. The columns are labeled as TIMP scores, Se concentration, Crude Coeff., Crude 95% CI, Crude P, Adjusted Coeff., Adjusted 95% CI, and Adjusted P. The rows are labeled as TIMP total, TIMP Observed, and TIMP Elicited, with each having three sub-rows for Lowest tertile, Middle tertile, and Highest tertile. The values in the table include coefficients, 95% confidence intervals, and P-values for both crude and adjusted models. Notable trends include no significant associations between maternal selenium concentration and TIMP scores across different tertiles and models.

Navigate back to Table 4.

## Table 5 Long description

The table presents the association between maternal plasma Se concentration and TIMP scores. It has three columns: TIMP scores, Se concentration, and two sets of data for Crude and Adjusted values, each with OR, 95% CI, and P values. The table has one row with the label Below average. Row 1: Below average, Crude OR 0.999, 95% CI 0.985, 1.014, P 0.94, Adjusted OR 0.999, 95% CI 0.986, 1.014, P 0.99.

Navigate back to Table 5.
